# *Dlg1* Knockout Inhibits Microglial Activation and Alleviates Lipopolysaccharide-Induced Depression-Like Behavior in Mice

**DOI:** 10.1007/s12264-021-00765-x

**Published:** 2021-09-06

**Authors:** Zhixin Peng, Xiaoheng Li, Jun Li, Yuan Dong, Yuhao Gao, Yajin Liao, Meichen Yan, Zengqiang Yuan, Jinbo Cheng

**Affiliations:** 1 Institute of Neuroscience, Hengyang Medical College, University of South China, Hengyang, 421001 China; 2 The Brain Science Center, Beijing Institute of Basic Medical Sciences, 27 Taiping Road, Haidian District, Beijing, 100850 China; 3 Beijing Institute for Brain Disorders, Capital Medical University, Beijing, 100069 China; 4 Institutes of Brain Sciences and Disease, Medical College, Qingdao University, Qingdao, 266071 China; 5 Center on Translational Neuroscience, College of Life and Environmental Science, Minzu University of China, Beijing, 100081 China

**Keywords:** Dlg1, Microglia, Neuroinflammation, Depression

## Abstract

Microglia-mediated neuroinflammation is widely perceived as a contributor to numerous neurological diseases and mental disorders including depression. Discs large homolog 1 (Dlg1), an adaptor protein, regulates cell polarization and the function of K^+^ channels, which are reported to regulate the activation of microglia. However, little is known about the role of Dlg1 in microglia and the maintenance of central nervous system homeostasis. In this study, we found that *Dlg1* knockdown suppressed lipopolysaccharide (LPS)-induced inflammation by down-regulating the activation of nuclear factor-κB signaling and the mitogen-activated protein kinase pathway in microglia. Moreover, using an inducible *Dlg1* microglia-specific knockout (*Dlg1*^*flox/flox*^; *CX3CR1*^*CreER*^) mouse line, we found that microglial *Dlg1* knockout reduced the activation of microglia and alleviated the LPS-induced depression-like behavior. In summary, our results demonstrated that Dlg1 plays a critical role in microglial activation and thus provides a potential therapeutic target for the clinical treatment of depression.

## Introduction

Major depressive disorder (MDD) is a severe psychiatric condition that affects nearly 15% of people worldwide [1], and it significantly increases the risk of suicide [2]. However, the pathophysiology of MDD is yet to be completely elucidated. Neuroinflammation is increasingly being accepted as a risk factor for emotional disorders, especially depression [3, 4]. Several clinical and preclinical studies have revealed that signatures of neuroinflammation, including activated microglia and inflammatory mediators, are commonly found in patients with depression [4, 5].

Microglia, the resident immune cells of the central nervous system (CNS), play indispensable roles in both health and disease [6]. Upon neuroinflammation, microglia are the first activated cells in the brain parenchyma [7]. They not only participate in the processes of neurodegenerative diseases [8–10] but are also involved in psychiatric disorders [11, 12]. Evidence has shown that microglial status is strongly associated with depression. Endotoxin stimulation can activate microglia and thus cause negative affective disorders [13], and microglia-derived pro-inflammatory cytokines are correlated with depression-like behaviors [14]. Furthermore, by targeting serotonin (5-HT), antidepressants inhibit depression-induced microglial activation and cytokine production, and alleviate depression-like symptoms [15–17]. Given that microglia-induced neuroinflammation is an important contributor to MDD, a major goal is to understand the precise underlying mechanism of microglial function in MDD.

Discs large homolog 1 (Dlg1, also known as SAP-97) is a member of the membrane-associated guanylate kinase family [18]. It is an adaptor protein that regulates cell polarity, neuronal Kv1 channel localization, and Kv1.3 channel function [19, 20]. Moreover, the Kv1.3 potassium channel has been reported to regulate the function of microglia, and selectively blocking Kv1.3 channels inhibits microglial activation [21]. In the CNS, Dlg1 interacts with AMPA (α-amino-3-hydroxy-5-methyl-4-isoxazolepropionic acid) and NMDA (N-methyl-D-aspartic acid) receptors when they are sorted and transported from the soma to synapses [22]. In the peripheral immune system, Dlg1 is involved in the activation of dendritic cells and T cells, and knockout of *Dlg1* inhibits the release of inflammatory cytokines [23, 24]. In addition, Dlg1 contributes to neuropsychiatric disorders, and is regarded as a risk factor for schizophrenia [25]. Despite the multiple roles of Dlg1, its roles in microglial activation and in MDD remain to be clarified.

In this study, we demonstrated that Dlg1 regulates microglial activation by targeting nuclear factor-κB (NF-κB) signaling and the mitogen-activated protein kinase (MAPK) pathway. *In vivo,* conditional knockout of microglial Dlg1 inhibited microglial activation, decreased inflammatory cytokine levels, and alleviated depression-like behaviors in mice, providing a potential therapeutic strategy to treat or slow down the progression of depression.

## Materials and Methods

### Mice

*CX3CR1*^*CreER*^ mice were purchased from the Jackson Laboratory (Bar Harbor, USA), and *Dlg1*^*flox/flox*^ mice were gifted by Dr. Wanli Liu (Tsinghua University, China). The generation of the *Dlg1*^*flox/flox*^ mouse has been described [26]. To generate microglia-specific knockout mice, homozygous *Dlg1*^*flox/flox*^ mice were crossed with mice expressing tamoxifen (TAM)-inducible Cre-recombinase under the control of the *CX3CR1* promoter (*CX3CR1*^*CreER*^ mice [27, 28]). Mice were given TAM by intragastric administration at the age of 6 weeks to induce microglia-specific knockout. All mice were housed in a specific pathogen-free environment at the Animal Care Facility in our institute. All animal experiments were approved by the Institutional Animal Care and Use Committee of the Beijing Institute of Basic Medical Sciences.

### Cell Culture and Transfection

All procedures were performed as previously described [29]. Briefly, BV2 and HEK293T cell lines were obtained from the American Type Culture Collection (Manassas, USA) and maintained in Dulbecco's modified Eagle's medium (Invitrogen, Waltham, USA) supplemented with 10% fetal bovine serum (Gibco, Grand Island, USA) and 1% penicillin-streptomycin (Invitrogen). All cells were maintained in a 5% CO_2_ atmosphere at 37°C.

For stable knockdown of *Dlg1* in BV2 cells, shRNAs against *Dlg1* (targeting sequence: #A: GTTGCATCATCTGTACTATTC, #B: GCAACCTCTTTCAGGCTTTAA) were used. Briefly, the shRNA was annealed and ligated into the pLKO.1 lentiviral vector (Addgene, Cambridge, USA) and then co-transfected with the viral packaging plasmids VSVG and ΔR812 into HEK293T cells. The viral supernatant was collected, centrifuged, filtered through a 0.45-μm filter, and then used to infected BV2 cells with polybrene (#sc-134220, Santa Cruz Biotechnology, Dallas, USA, 1:1000). Three days after infection, the cells were selected with puromycin.

### Plasmid Constructs and Selection of Cells with Stable Expression

Full-length *Dlg1* was amplified from a mouse cDNA library by PCR and inserted into the pCDH-CMV-MCS-EF1-Puro expression vector (Sigma-Aldrich, St Louis, USA) using the *EcoRI* and *NotI* restriction sites. To generate cells that stably overexpressed *Dlg1*, BV2 cells were infected with the retroviral vector pCDH encoding *Dlg1* and selected with puromycin.

### Reverse Transcription and Real-Time PCR

Total RNA was extracted from cultured cells, brain tissue, or sorted cells using TRIzol^TM^ reagent (#15596018, Invitrogen). cDNA was obtained using a one-step first strand cDNA synthesis kit (AE311-03, TransGen Biotech, Beijing, China). The primer sequences used were as follows:

- *GAPDH* forward: 5′-AGGTCGGTGTGAACGGATTTG-3′;
- *GAPDH* reverse: 5′-TGTAGACCATGTAGTTGAGGTCA-3′;
- *Dlg1* forward: 5′-AGTGACGAAGTCGGAGTGATT-3′;
- *Dlg1* reverse: 5′-GTCAGGGATCTCCCCTTTATCT-3′;
- *TNF-α* forward: 5′-CCCTCACACTCAGATCATCTTCT-3′;
- *TNF-α* reverse: 5′-GCTACGACGTGGGCTACAG-3′;
- *Il-6* forward: 5′-CCAAGAGGTGAGTGCCTTCCC-3′;
- *Il-6* reverse: 5′-CTGTTGTTCAGACTCTCTCCCT-3′;
- *Il-1β* forward: 5′-GCAACTGTTCCTGAACTCAACT-3′;
- *Il-1β* reverse: 5′-ATCTTTTGGGGTCCGTCAACT-3′.

### Drugs and Treatment

Lipopolysaccharide (LPS; Sigma-Aldrich) was dissolved in saline (0.9% NaCl), and injected intraperitoneally at 1 mg/kg. Tamoxifen (#S1238, Selleckchem, Houston, USA) was dissolved in corn oil. Mice at the age of 6 weeks were given a total dose of 20 mg tamoxifen intragastrically for three consecutive days to induce microglial *Dlg1*-specific knockout.

### Western Blotting

Whole cells and hippocampal tissue were lysed on ice using cell lysis buffer [50 mmol/L Tris-HCl (pH 7.4), 150 mmol/L NaCl, 1 mmol/L EDTA, 1% Triton X-100, 0.1% deoxycholate, 0.05% sodium dodecyl sulfate (SDS), and protease inhibitor cocktail]. The lysates were centrifuged at 15,000× g for 15 min at 4°C, then supernatants were mixed with 6× SDS loading buffer and boiled for 10 min. Samples were separated by SDS-PAGE and transferred to a nitrocellulose (NC) membrane which was blocked in 5% milk and subsequently incubated overnight with the primary antibody. Relevant horseradish peroxidase (HRP)-conjugated secondary antibodies were incubated, and an electrochemiluminescence (ECL) detection reagent was applied to the NC membrane. The protein signal was detected using an X-Ray film processor (Optimax, New York, USA). The antibodies used for immunoblotting were as follows: anti-SAP97 (K64/15) (#75-030, Antibodies Incorporated, Davis, USA), anti-iNOS/NOS Type II (#610332, BD Biosciences, San Jose, USA), anti-Phospho-IKKα/β (S176/180, 16A6) (#2697P), anti-p38 MAPK (#9212), anti-phospho-p38 MAPK (Thr180/Tyr182) (#4511), anti-JNK2 (56G8) (#9258), anti-phospho-SAPK/JNK (Thr183/Tyr185) (#9251), anti-IκBα (44D4) (#4812), and anti-phospho-IκBα (Ser32) (#2859) (Cell Signaling Technology, Beverly, USA), anti-IKKα (CHUK) (#A2062, ABclonal Technology, Wuhan, China), anti-GAPDH (#CW0266A, CWBiotech, Beijing, China), and anti-β-actin (60008-1-Ig, Proteintech Group, Campbell Park, Chicago, USA).

### Dual-Luciferase Reporter System

The NF-κB reporter was generated in our laboratory. Briefly, the NF-κB promoter was cloned into a pGL3-luciferase reporter vector (Promega, Madison, USA). The pCMV-*Renilla* plasmid and NF-κB reporter were co-transfected into HEK293T cells using Lipofectamine 2000 transfection reagent (#11668019, Invitrogen). Sixteen hours after transfection, the cells were lysed and luciferase activity was measured using a dual-luciferase reporter detection system (Promega).

### Immunohistochemistry and Immunofluorescence

After anesthesia (0.7% pentobarbital sodium intraperitoneal injection), each mouse was perfused with normal saline and the brain was removed, fixed in 4% paraformaldehyde for 24 h, and dehydrated overnight in 30% sucrose in phosphate buffered saline (PBS). The whole brain was embedded in optimal cutting temperature compound (OCT) and sectioned on a freezing microtome (CM3050S, Leica, Wetzlar, Germany). The coronal sections were incubated overnight with anti-goat IBA1 antibody (1:500; Cat. 019-19741, WAKO, Japan) and anti-mouse GFAP antibody (1:400; MAB360, Millipore, Darmstadt, Germany). Immunostaining was visualized with DAB kit (Zhongshanjinqiao, Beijing, China) after reaction with hydrogen peroxide. All stained sections were examined under a laser scanning confocal microscope (A1, Nikon, Tokyo, Japan).

### Open Field Test (OFT)

The mice were kept in the test room for 2 h in advance for preconditioning. During the experiment, each mouse was placed in a 32 × 32 cm^2^ test box. The test program was started to record the spontaneous activity of the mouse during 5 min and to count the total distance travelled and the time spent in the central area.

### Elevated Plus Maze (EPM)

The mice were kept in the test room for 2 h in advance for preconditioning. During the test, each mouse was gently placed in the center area facing the open arm. The recording software was then started to record the activity time and the number of entries into the open arm during 5 min.

### Tail Suspension Test (TST)

The mice were kept in the test room for 2 h in advance for preconditioning. We stuck medical tape to the tail tip, and the mouse was hung on the instrument with a clip. The experimental time was 6 min and the immobility time of each mouse was recorded in the last 4 min of the test.

### Forced Swimming Test (FST)

The day before the experiment, the mice were allowed to swim in water for 5 min. On the day of the experiment, the mice were kept in the test room for preconditioning. During the test, each mouse was placed in a beaker (volume 3 L) filled with water at 18–21 °C. The total test time was 6 min. The immobility time was recorded during the last 4 min of the test.

### Statistical Analysis

Data are expressed as the mean ± SEM from at least two independent experiments. Statistical differences between the test and control values were analyzed using Student’s *t*-test. For multiple comparisons, statistical differences were analyzed by applying ordinary one-way ANOVA (Tukey’s multiple comparison test). Data were considered significant as follows: *P < 0.05, **P < 0.01, and ***P < 0.001. Statistical analysis was performed using GraphPad Prism (version 6.07, GraphPad, San Diego, USA).

## Results

### Knockdown of *Dlg1* in Microglia Inhibits LPS-induced Inflammation

**Fig. 1:**
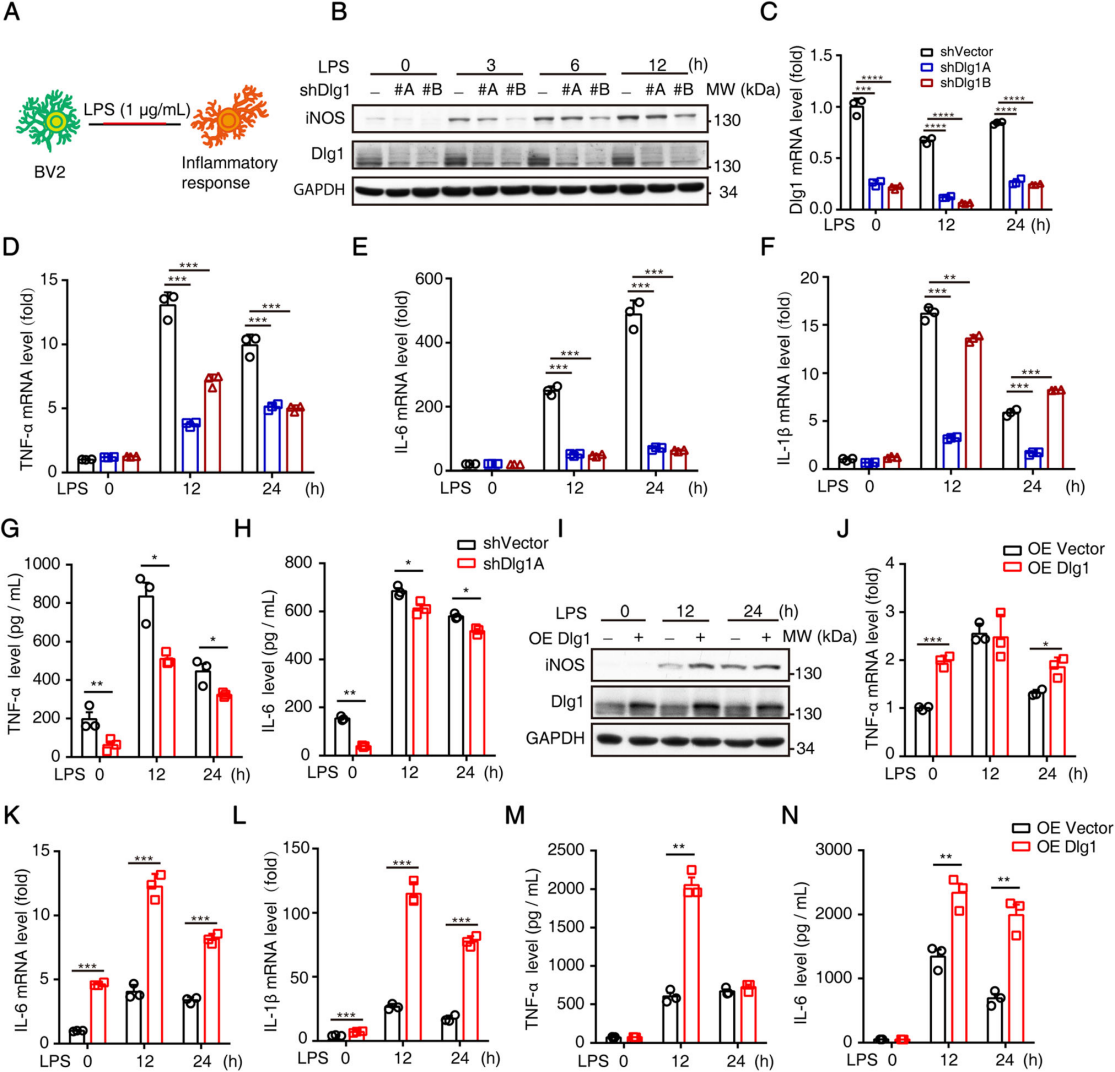
Microglial *Dlg1* knockdown inhibits LPS-induced inflammation. **A** Cartoon of LPS stimulation in BV2 cells. **B** Immunoblots of iNOS, Dlg1, and GAPDH proteins in control and *Dlg1*-knockdown microglial cells after exposure to LPS (1 µg/mL) for the indicated times. **C–F** RT-PCR analysis of *Dlg1*, *TNF-α*, *IL-1β*, and *IL-6* mRNA levels in control and *Dlg1*-knockdown microglial cells after exposure to LPS (1 µg/mL) for the indicated times. **G, H** ELISA analysis of secreted TNF-α and IL-6 levels in control and *Dlg1*-knockdown BV2 cells after exposure to LPS (1 µg/mL) for the indicated times. **I** Immunoblots of iNOS, Dlg1, and GAPDH proteins in control and *Dlg1*-overexpressing microglial cells after exposure to LPS (1 µg/mL) for the indicated times. **J**–**L** RT-PCR analysis of *TNF-α*, *IL-1β*, and *IL-6* mRNA levels in control and *Dlg1*-overexpressing microglial cells. At least three independent experiments were carried out. **M, N** ELISA analysis of secreted TNF-α and IL-6 levels in control and *Dlg1*-overexpressing BV2 cells after exposure to LPS (1 µg/mL) for the indicated times. Data are presented as the mean ± SEM; *P <0.05, **P <0.01, ***P <0.001, two-tailed unpaired Student’s *t*-test.

LPS is a commonly-used agonist that induces the activation of microglia. This process is characterized by the overwhelming release of pro-inflammatory cytokines [30, 31]. To investigate the role of Dlg1 in microglial activation, we exposed BV2 cells with *Dlg1* knockdown to LPS (1 μg/mL) at different time points (Fig. 1A). Western blot analysis showed that the iNOS protein level was significantly lower in *Dlg1*-knockdown cells than in control cells (Fig. 1B, C). In addition, knockdown of *Dlg1* inhibited the up-regulation of pro-inflammatory cytokines, including tumor necrosis factor (TNF-α), interleukin-1 beta (IL-1β), and IL-6 (Fig. 1D–H). These results suggest that Dlg1 is involved in LPS-induced inflammation, and knockdown of *Dlg1* in microglia significantly reduces inflammatory cytokine levels.

To support our findings, we overexpressed *Dlg1* in BV2 cells, and these cells were also exposed to LPS at different time points. Consistently, both the iNOS protein level and the cytokine levels were significantly increased when *Dlg1* was overexpressed (Fig. 1I–N). Taken together, these results indicate that Dlg1 plays an important role in microglia-mediated inflammation.

### *Dlg1* Participates in Microglia-mediated Inflammation by Targeting NF-κB Signaling and the MAPK Pathway

**Fig. 2:**
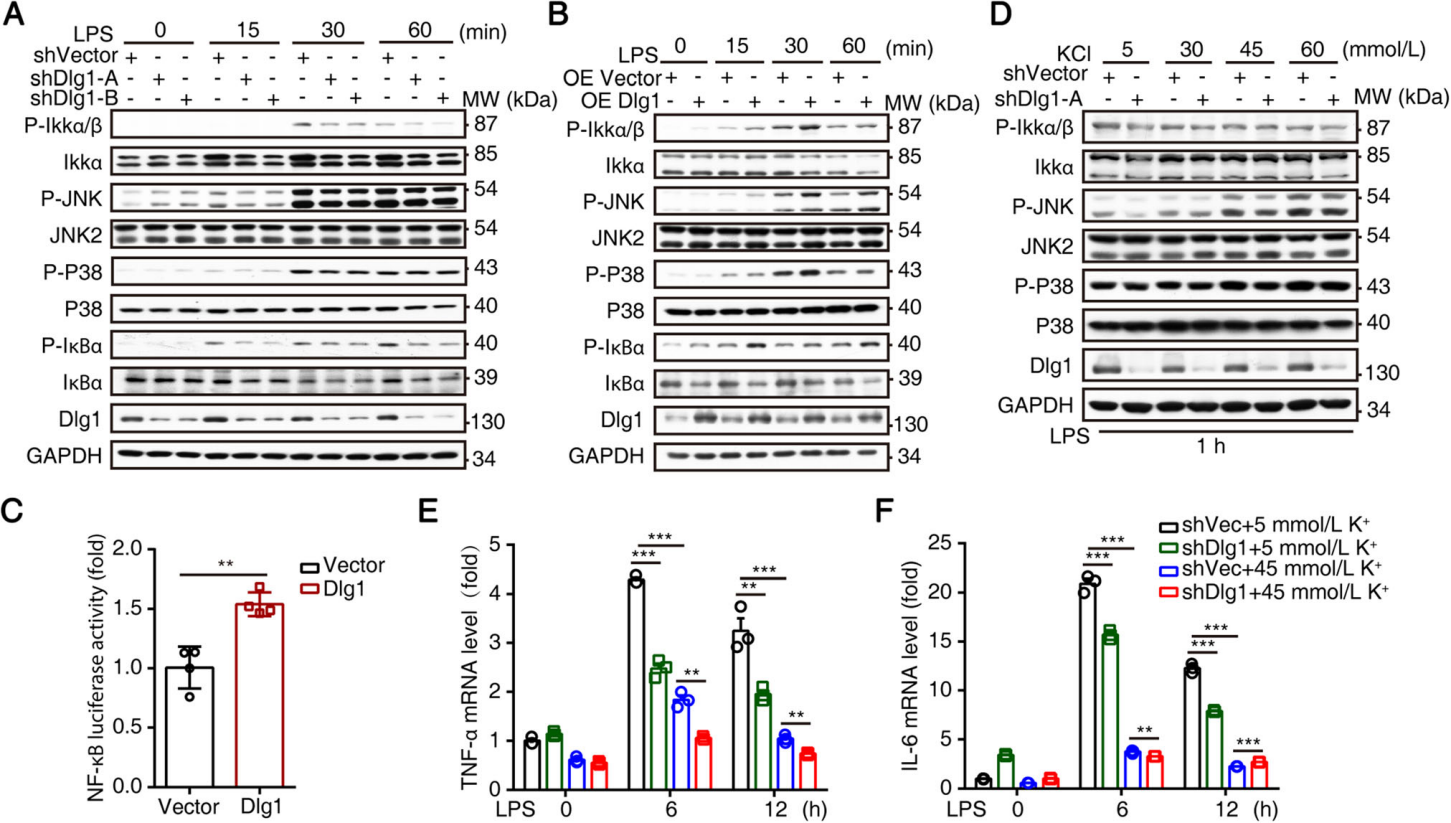
Dlg1 regulates the NF-κB signaling pathway in microglia. **A**, **B** Immunoblots of P-IKKα/β, IKKα, P-JNK, JNK2, P-P38, P38, P-IκBα, IκBα, Dlg1, and GAPDH proteins in control, *Dlg1*-knockdown (**A**) and *Dlg1*-overexpressing (OE; **B**) microglial cells after exposure to LPS (1 µg/mL) for the indicated times. **C** Quantitative analysis of the effect of *Dlg1* overexpression on NF-κB luciferase activity. **D** Immunoblots of P-IKKα/β, IKKα, P-JNK, JNK2, P-P38, P38, Dlg1, and GAPDH proteins in control and *Dlg1*-knockdown microglial cells after exposure to LPS (1 µg/mL, 60 min) and KCl (5, 30, 45, and 60 mmol/L). **E**, **F** RT-PCR analysis of *TNF-α* and *IL-6* mRNA levels in control and *Dlg1*-knockdown microglia. Data are presented as the mean ± SEM; *P < 0.05, **P < 0.01, ***P < 0.001, two-tailed unpaired Student’s *t*-test.

It has been well corroborated in previous studies that LPS is an activator of NF-κB signaling and the MAPK pathway [30, 32]. To study the mechanism underlying Dlg1 and microglia-mediated inflammation, we used a short-term LPS challenge, and found that the phosphorylation levels of various pathway proteins declined markedly in *Dlg1*-knockdown BV2 cells, including P-IKKα/β, P-IκBα, P-JNK, and P-P38 (Fig. 2A). Correspondingly, overexpression of *Dlg1* significantly increased the levels of these proteins (Fig. 2B). These results suggest that Dlg1 participates in microglia-mediated inflammation by regulating NF-κB signaling and the MAPK pathway. Since alterations in Dlg1 affect the protein levels of NF-κB signaling, we then asked whether Dlg1 affects its activation. Using a dual-luciferase reporter system, we found that the NF-κB promoter luciferase showed enhanced activity upon overexpression of *Dlg1* (Fig. 2C). Collectively, these results indicate that Dlg1 regulates NF-κB signaling and the MAPK pathway and thus plays an important role in microglia-mediated inflammation.

Previous studies have shown that Dlg1 is an adaptor protein of the Kv1.3 potassium channel by ensuring K^+^ outflow and subsequent Ca^2+^ inflow events [22, 23]. To determine whether Dlg1 regulates microglia-mediated inflammation depending on the activity of K^+^ channels, we applied a serial concentration gradient of K^+^ culture medium to inhibit K^+^ channel activity [33] and found that the decreased levels of p-IKKα/β, P-JNK, and P-P38 regulated by *Dlg1* knockdown diminished as the concentration of K^+^ increased (Fig. 2D). Furthermore, the levels of the inflammatory cytokines TNF-α and IL-6 were significantly inhibited when the K^+^ channel activity was blocked by high concentrations of K^+^ (Fig. 2E, F). Together, these results indicate that Dlg1 regulates microglia-mediated inflammation in a K^+^-dependent manner.

### Microglial *Dlg1* Knockout Alleviates Depression-like Behavior

**Fig. 3:**
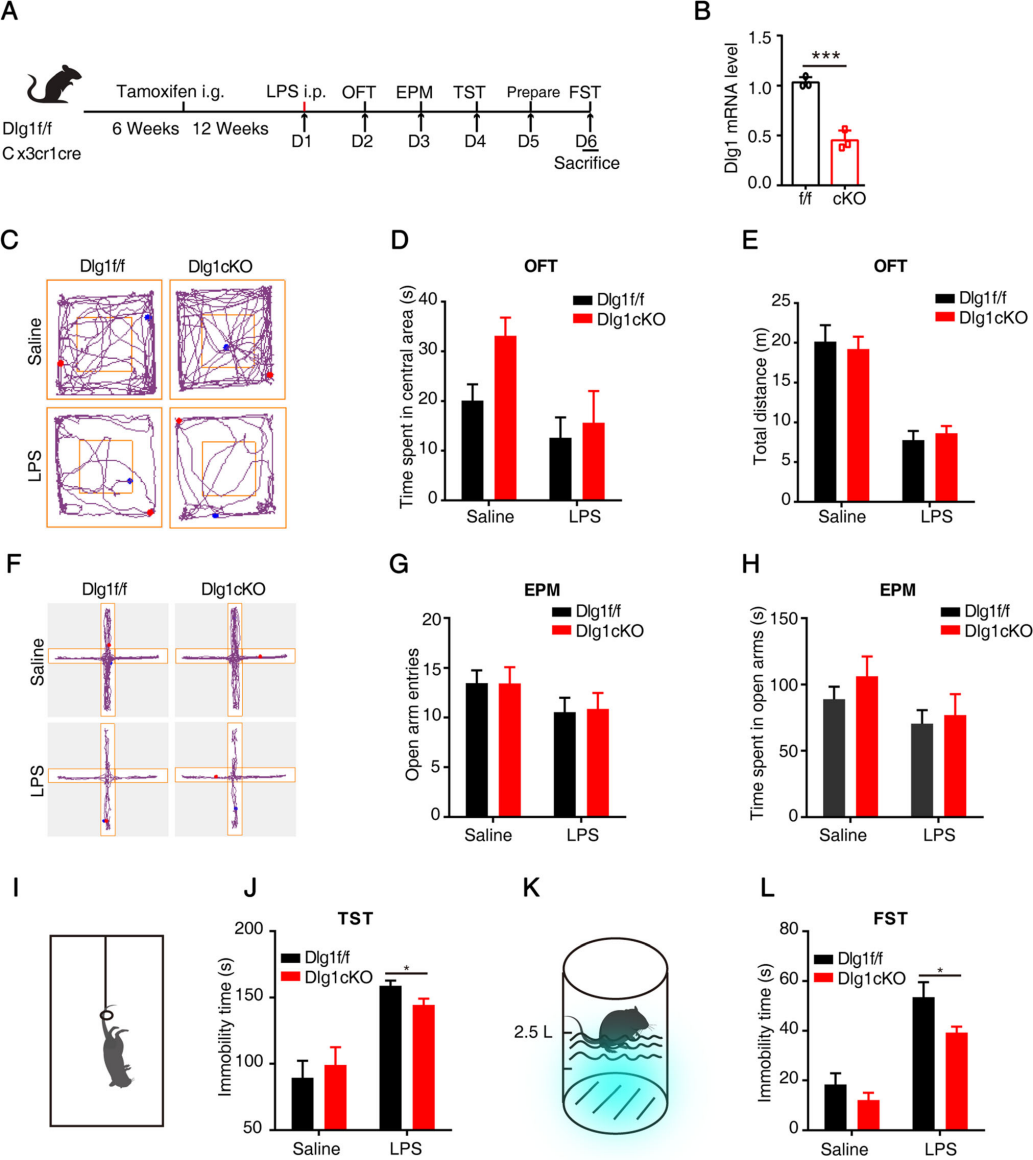
Microglial *Dlg1* knockout alleviates LPS-induced depression-like behavior in mice. **A** Timeline of drug administration and behavioral tests. **B** RT-PCR analysis of microglial *Dlg1* mRNA levels from *Dlg1*^*flox/flox*^ (*n* = 3) and cKO (*n* = 3) mice. **C** Representative images showing the activity trajectories of mice in the open field test (OFT). **D** Time spent in the central area by *Dlg1*^*flox/flox*^ (*n* = 11) and cKO (*n* = 7) mice in the OFT 24 h after saline or LPS (1 mg/kg) injection. **E** Total distance travelled in the OFT by *Dlg1*^*flox/flox*^ (*n* = 11) and cKO (*n* = 7) mice. **F** Representative images showing the activity trajectories of mice in the elevated plus maze (EPM). **G**, **H** Numbers of entries into open arms (**G**) and time spent in the center (**H**) in the EPM. **I** Cartoon of the tail suspension test (TST). **J** Immobility times in the TST. **K** Cartoon of forced-swimming test (FST). **L** Immobility times in the FST. Data are presented as the mean ± SEM; *P < 0.05; **P < 0.01, ***P < 0.001, two-tailed unpaired Student’s *t*-test.

Given that *Dlg1* knockdown significantly reduced the inflammatory response in microglia, we next sought to explore the role of Dlg1 in depression. We generated mice with inducible microglia-specific knockout of *Dlg1* (*Dlg1*^*flox/flox*^; *CX3CR1*^*CreER*^, abbreviated as cKO mice), and used tamoxifen to induce Cre-recombinase activity in microglia (Fig. 3A). Compared to *Dlg1*^*flox/flox*^ mice, the Dlg1 level in cKO mice decreased significantly (Fig. 3B). The LPS-induced depression model has been commonly used in studies based on the causal link between neuroinflammation and depression [34–36]. In our experiments, we applied an LPS challenge after tamoxifen administration to induce depression-like behavior in *Dlg1*^*flox/flox*^ and cKO mice as assessed by behavioral tests (Fig. 3A). As shown in Fig. 3C–H, treatment with LPS decreased the center time and total distance in the OFT, and decreased the open arm entries and time in the EPM. However, cKO mice did not show significant differences from *Dlg1*^*flox/flox*^ mice, suggesting that microglial *Dlg1* knockout does not impair LPS-induced anxiety-like behaviors.

However, in the TST, cKO mice had a shorter immobility time than *Dlg1*^*flox/flox*^ mice, suggesting that microglial *Dlg1* knockout alleviates the depression-like behavior (Fig. 3I, J). To further characterize the role of Dlg1, we used the FST. Consistent with the results in the TST, microglial *Dlg1* knockdown significantly decreased the immobility time, suggesting an improvement in depression-like behavior (Fig. 3K, L). Together, these results demonstrate that deletion of microglial Dlg1 alleviates inflammation-induced depression-like behaviors in mice.

### *Dlg1* Knockout Inhibits Microglial Activation *In Vivo*

**Fig. 4:**
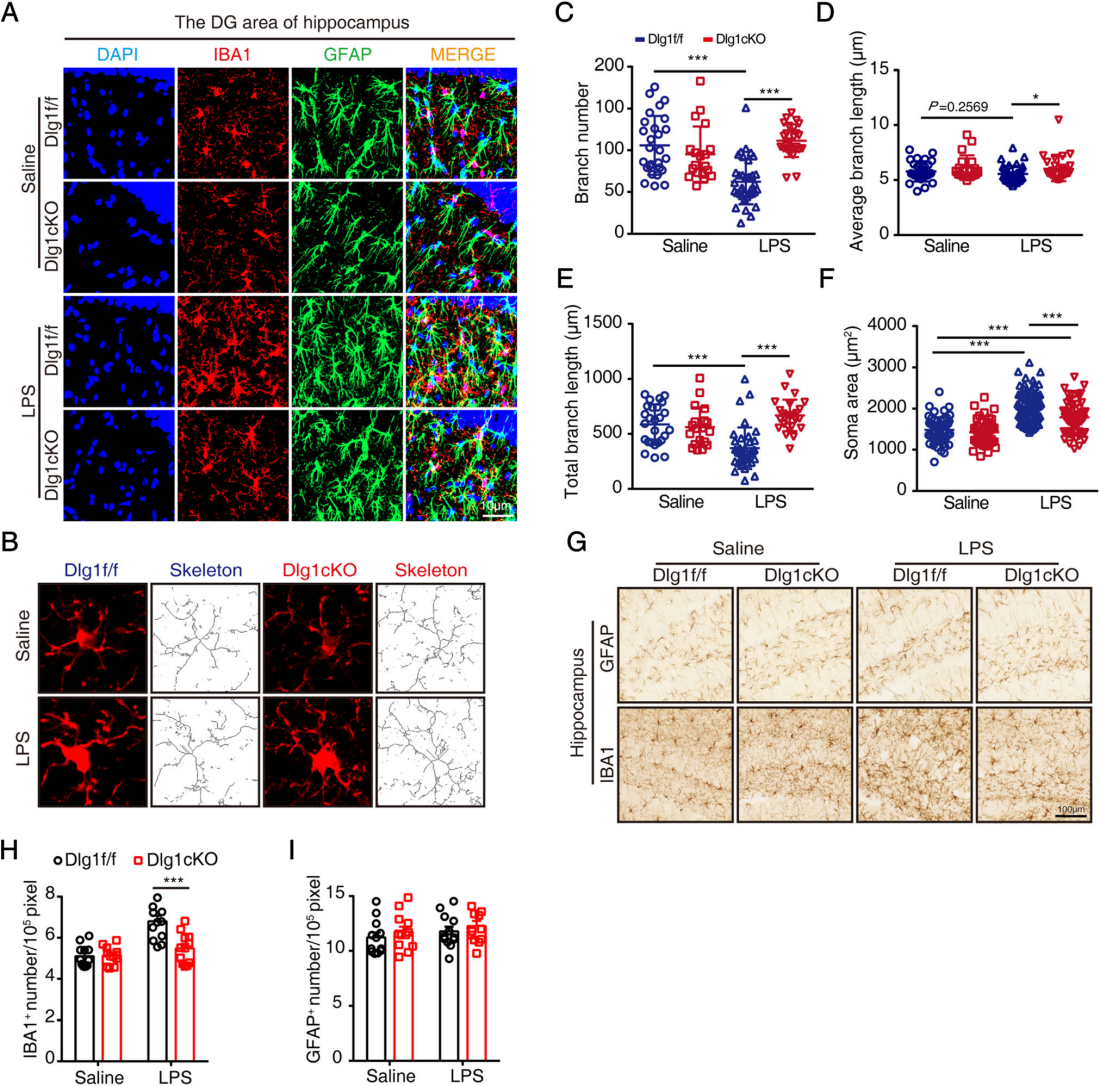
Microglial *Dlg1* knockout impedes microglial activation *in vivo*. **A** Immunofluorescent staining of IBA1 and GFAP in the hippocampal DG area of *Dlg1*^*flox/flox*^ and cKO mice with or without LPS exposure (1 mg/kg) (scale bar, 10 μm). **B** Images and schematics of microglial skeleton analysis. **C**–**F** Quantitative analysis of number of microglial branches (**C**), average branch length (**D**), total branch length (**E**), and soma area (**F**). **G** Immunohistochemical staining of IBA1 and GFAP in the hippocampus of *Dlg1*^*flox/flox*^ and cKO mice (scale bar, 100 μm). **H**, **I** Quantitative analysis of IBA1-positive (**H**) and GFAP-positive cell numbers (**I**). Data are presented as the mean ± SEM; *P < 0.05, **P < 0.01, ***P < 0.001, two-tailed unpaired Student’s *t*-test.

We next investigated why *Dlg1* knockout alleviates depression-like behavior and how microglia change in cKO mice. To address this, we performed IBA1 immunofluorescence staining and found that microglia were robustly activated in the brain of mice injected with LPS, including changes in density and morphology (Fig. 4A, B). Microglial *Dlg1* knockout significantly rescued the LPS-induced reduction in branch number and length, as well as increasing the soma area (Fig. 4C–F). Furthermore, LPS injection increased microglial numbers in the hippocampus of *Dlg1*^*flox/flox*^ mice, and *Dlg1* microglial conditional knockout significantly reduced this increase (Fig. 4G, H), with no effect on astrocyte number (Fig. 4G, I). These results suggest that *Dlg1* knockout in microglia markedly inhibits microglial activation in the mouse brain.

**Fig. 5:**
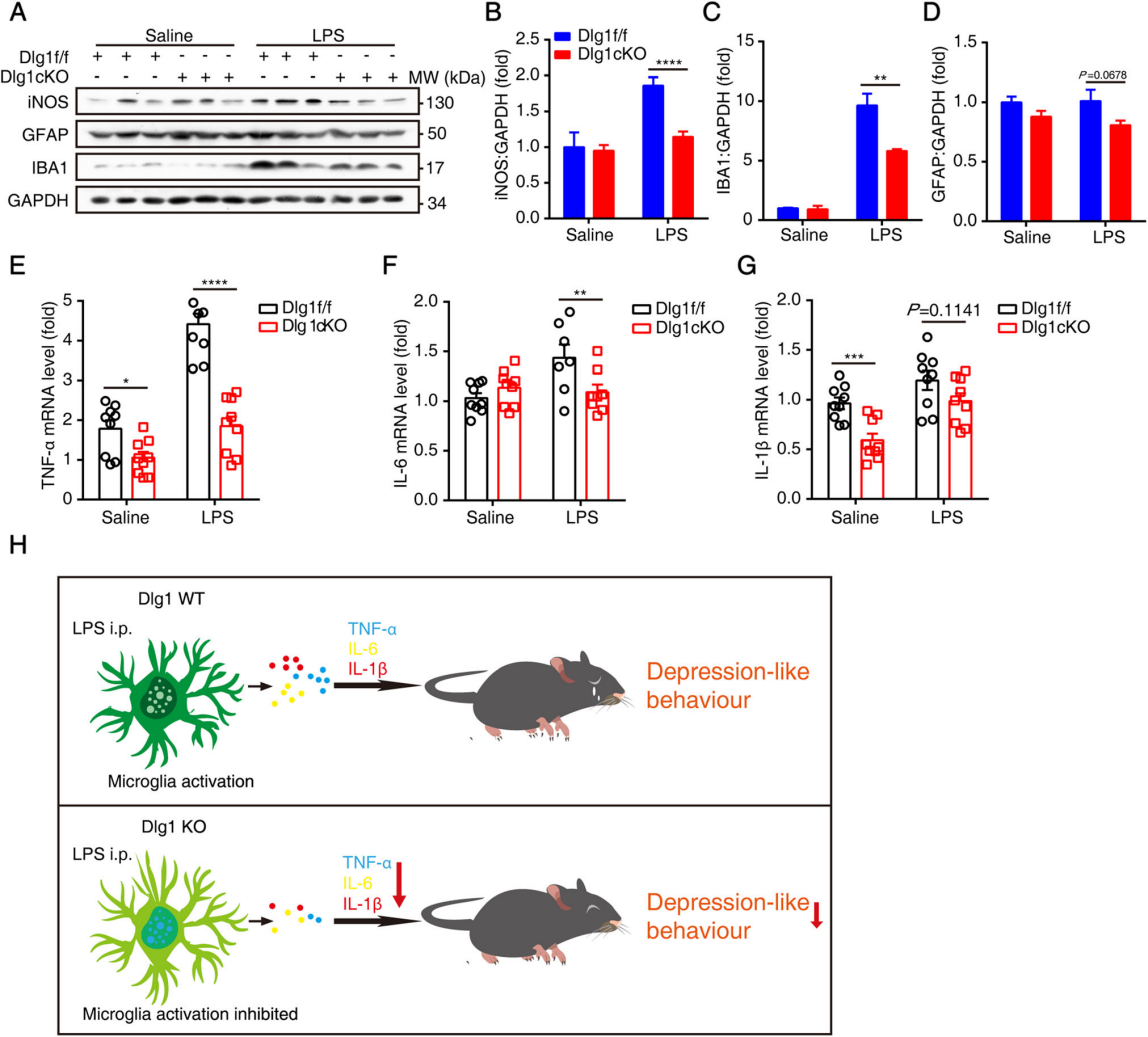
Microglial *Dlg1* knockout decreases inflammation in the mouse brain. **A** Immunoblots of iNOS, GFAP, IBA1, and GAPDH proteins in the hippocampus of *Dlg1*^*flox/flox*^ and cKO mice. **B–D** Quantitative analysis of iNOS, IBA1, and GFAP protein levels normalized to GAPDH. **E–G** RT-PCR analysis of *TNF-α*, *IL-1β*, and *IL-6* mRNA levels in the hippocampus of *Dlg1*^*flox/flox*^ and cKO mice. **H** Model showing the role of Dlg1 in inflammation-induced depression-like behaviors in mice. Data are presented as the mean ± SEM; *P < 0.05, **P < 0.01, ***P < 0.001, two-tailed unpaired Student’s *t*-test.

To determine whether inflammatory cytokines also declined, we measured the protein levels of iNOS and IBA1 in the hippocampus and found that upon LPS challenge they were significantly reduced in cKO mice, with a decreasing trend of GFAP levels compared with *Dlg1*^*flox/flox*^ mice (Fig. 5A–D). Moreover, we found that the inflammatory cytokines TNF-α and IL-6 were significantly reduced in cKO mice (Fig. 5E, F), with a decreasing trend of IL-1β levels (Fig. 5G).

These results collectively indicate that microglial conditional knockout of *Dlg1* inhibits microglial activation, and thus alleviates the depression-like behavior induced by neuroinflammation (Fig. 5H).

## Discussion

As a representative of bacterial infection, systemic LPS injection (1 mg/kg) causes time-dependent behavioral changes in mice. Pro-inflammatory cytokines in the blood peak at ~2 h, and depression-like behaviors occur 24 h later [14, 37–39]. In this study, we used LPS injection to create a depression-like mouse model. Microglial *Dlg1* deletion ameliorates the symptoms of depression in mice, and the hippocampus is relatively vulnerable to acute inflammatory attack during depression [34]. Our results demonstrated that *Dlg1* knockout reduced microglial activation and inflammatory cytokine levels in the hippocampus, suggesting that Dlg1 functions as an important regulator of inflammation-induced depression.

Neuroinflammation is involved in the regulation of motivational and emotional states in various pathological processes. The increased expression and release of inflammatory mediators, generally manifested as pro-inflammatory cytokines, acute phase proteins, chemokines, and adhesion molecules, are related to human depressive symptoms and negative affective states in mice [40–45]. Elevated levels of IL-6, IL-1β and TNF-α are found in both peripheral blood and cerebrospinal fluid [40, 46–49]. Moreover, the concentrations of these cytokines in plasma are positively correlated with the severity of depressive symptoms [41, 50]. Furthermore, previous studies have revealed that functional allelic variants of the *IL-1β* and *TNF-α* genes increase the risk of depression and are associated with decreased responsiveness to antidepressant therapy [51, 52]. Mechanistically, the increased pro-inflammatory cytokines mediated by NF-κB signaling [53] cause long-term alterations in neuropeptide and neurotransmitter synthesis, leading to metabolic changes in serotonin, dopamine, and other related neurotransmitters [54, 55]. Moreover, the MAPK pathway is associated with dysregulation of the hypothalamic–pituitary–adrenal axis, one of the strongest biological correlates of MDD [56, 57]. Here, we found that the NF-κB signaling and MAPK pathways were involved in microglia-mediated inflammation. Knockout of *Dlg1* in microglia inhibited the activation of the NF-κB signaling and MAPK pathways, and markedly alleviated depression-like behaviors. Numerous studies have revealed that voltage-gated K^+^ channels are involved in microglial activation [58, 59]. In this study, we found that the effects of Dlg1 were partially rescued by incubation with high concentrations of K^+^, suggesting that K^+^ flux is involved in the effect of Dlg1 in microglia. However, the target and the regulatory mechanism of Dlg1 in microglial activation need to be further investigated.

Microglia are resident immune cells of the CNS. Our recent studies showed that inhibition of microglial activation-induced neuroinflammation alleviates brain injury in multiple neurodegenerative diseases and psychological disorders [9, 10, 60, 61]. Moreover, a recent study showed that microglial activation regulates negative emotions through prostaglandin-dependent striatal neurons, and thus plays a key role in the development of major depression [62]. In this study, we found that knockout of *Dlg1* in microglia alleviated neuroinflammation-induced depression-like behaviors in mice, providing a novel way to interfere with microglial activation and an alternative treatment strategy for depression.

## Conclusions

In summary, our study demonstrated that knockdown of *Dlg1* inhibits the activation of NF-κB signaling and the MAPK pathway in microglia, and consequently reduces microglial activation and the release of pro-inflammatory cytokines. Furthermore, deletion of microglial *Dlg1* alleviates inflammation-induced depression-like symptoms in mice. Our findings demonstrate that Dlg1 plays a vital role in microglial activation and provides a potential therapeutic target for the treatment of depression.
